# The clinical efficacy of arthroscopic therapy with knee infrapatellar fat pad cell concentrates in treating knee cartilage lesion: a prospective, randomized, and controlled study

**DOI:** 10.1186/s13018-021-02224-9

**Published:** 2021-01-28

**Authors:** Yiqin Zhou, Haobo Li, Dong Xiang, Jiahua Shao, Qiwei Fu, Yaguang Han, Jun Zhu, Yi Chen, Qirong Qian

**Affiliations:** Department of Joint Surgery and Sports Medicine, Shanghai Changzheng Hospital, Naval Medical University, No.415 Fengyang Road, Shanghai, 200003 China

**Keywords:** Knee cartilage lesion, Randomized controlled trial, Infrapatellar fat pad adipose tissue, Mesenchymal stromal cells, Cartilage regeneration

## Abstract

**Introduction:**

To evaluate the clinical efficacy of arthroscopic therapy with infrapatellar fat pad cell concentrates in treating knee cartilage lesions, we conducted a prospective randomized single-blind clinical study of controlled method.

**Methods:**

Sixty cases from Shanghai Changzheng Hospital from April 2018 to December 2019 were chosen and randomly divided into 2 groups equally. Patients in the experiment group were treated through knee arthroscopy with knee infrapatellar fat pad cell concentrates containing mesenchymal stromal cells, while patients in the control group were treated through regular knee arthroscopic therapy. VAS and WOMAC scores were assessed at pre-operation, and 6 weeks, 12 weeks, 6 months, and 12 months after intervention. MORCART scores were assessed at pre-operation and 12 months after intervention.

**Results:**

Twenty-nine cases in the experiment group and 28 cases in the control group were followed up. No significant difference in VAS, WOMAC, and MOCART scores were found between the two groups before surgery (*P* > 0.05). The WOMAC total and WOMAC function scores of the experiment group were significantly lower than those of the control group 6 months and 12 months after surgery (*P* < 0.05). The VAS rest and VAS motion scores of the experiment group were found significantly lower than those of the control group 12 months after surgery (*P* < 0.05). The MOCART scores of the experiment group were found significantly higher compared with the control group 12 months after surgery (*P* < 0.05). No significant difference in WOMAC stiffness scores were found between the two groups.

**Conclusions:**

The short-term results of our study are encouraging and demonstrate that knee arthroscopy with infrapatellar fat pad cell concentrates containing mesenchymal stromal cells is safe and provides assistance in reducing pain and improving function in patients with knee cartilage lesions.

**Trial registration:**

ChiCTR1800015379. Registered on 27 March 2018, http://www.chictr.org.cn/showproj.aspx?proj=25901.

## Introduction

Articular cartilage lesions of the knee are extremely common, with a high morbidity rate of 5% in the general population and 22–50% in the athlete population [1, 2]. They can be caused by trauma, malalignment in the load-bearing axis, and aging. Currently, traditional clinical therapies for knee cartilage lesions include drug therapy (with glucosamine, for example), simple arthroscopic debridement, microfracture surgery, and autologous osteochondral transplantation [3–7]. However, the therapeutic efficacy of drug therapy and simple arthroscopic debridement is limited [1]. In addition, the utility of the microfracture technique is confined to the treatment of small-scale damage, and the use of autologous osteochondral transplantation is limited by an insufficient donor supply [7–10]. Currently, these known therapies can be effective for a short period, but their long-term efficacies are uncertain [11, 12].

Recently, cartilage tissue engineering based on mesenchymal stromal cells (MSCs) has become an effective treatment choice [12–14]. MSCs are a kind of multipotent stromal cells, which may have potential as a treatment option for early cartilage lesions. The infrapatellar fat pad (IPFP), routinely removed in knee arthroscopic surgery, is a good source of MSCs. Compared with other tissues, the use of the IPFP, which is a waste source in regular knee arthroscopy surgery, can result in less damage to the donor zone and offer more MSCs with differentiative capacity [15–22].

Stromal cell therapy, which can be categorized into pure cell and cell concentrate therapies [21], is still being investigated in clinical trials. Compared with pure cell therapy, treatment with cell concentrates containing stromal cells takes less time in the cell isolating procedure and can be more expediently [19, 20]. In our study, we conducted a randomized controlled trial to evaluate whether knee arthroscopic therapy with IPFP cell concentrates containing MSCs is safe and can improve clinical symptoms of patients with knee cartilage lesions.

## Methods

### Trial design

This study is a randomized controlled trial of Evidence Level I from April 2018 to December 2019. The study conforms to the principles of the Declaration of Helsinki. The trial is endorsed by the Shanghai Changzheng Hospital Ethics Committee (CZEC (2016)-02). There is no commercial sponsorship of this study. The trial is registered (ChiCTR1800015379).

### Trial participants

All participants learned about research recruitment from outpatient surgeons. To be admitted to the trial, the inclusion criteria for patients were (1) age between 18 and 75 years old, (2) presence of clearly indicated articular cartilage lesions on MRI and Kellgren-Lawrence grade ≤ level 3, (3) obvious knee pain or discomfort lasting for more than 3 months, (4) understanding of the treatment and signed informed consent by the patient or their family, (5) articular cartilage lesions diagnosed by arthroscopy without targeted treatment.

The exclusion criteria for patients were (1) previous surgical procedures for articular cartilage lesions, (2) history of intra-articular injection or history of peri-articular invasive treatments within 3 months of the start of the study, (3) symptoms and imaging findings localized in the patellofemoral joint, (4) presence of malignant neoplasms, (5) active infection anywhere in the body, (6) pregnant or lactating women or women preparing for pregnancy, (7) articular cartilage lesions caused by infectious or gouty arthritis, (8) presence of autoimmune diseases such as rheumatoid arthritis or ankylosing spondylitis, (9) diabetes with fasting blood glucose over 8 mmol/L, (10) general poor health and not recommended for surgery, (11) presence of severe diseases such as cerebral hemorrhage, severe pneumonia, or multiple organ dysfunction, (12) failure to give consent to participate in the study, (13) diagnosis of Charcot joint, (14) findings that may increase patient risk or influence the results of the intervention, (15) other reasons for not being fit for the study.

After being advised about the trial protocol and prior to the start, all patients submitted written informed consent.

### Randomization and blinding

Randomization was performed by an independent researcher not involved in the treatment or outcome measurement. After providing informed consent, each eligible patient was randomly assigned a sequence number from a computer-generated random number list and randomly separated into one of the two study groups: an experiment group (knee arthroscopic therapy with autologous IPFP cell concentrates) or a control group (knee arthroscopic therapy only). The random number was concealed in a sealed envelope and saved by a researcher who did not participate in the trial. The final unblinding was performed after data collection. Participants were kept blinded to the allocated treatment during the follow-up period. All participants in the experiment group and control group received knee arthroscopic therapy by the same surgeon. Outcome assessors were blinded to the study groups and did not take part in the implementing interventions. During follow-up, all the doctors, radiologists, and statisticians were unaware of the study group assignments.

### Interventions

#### Experiment group

**Fig. 1:**
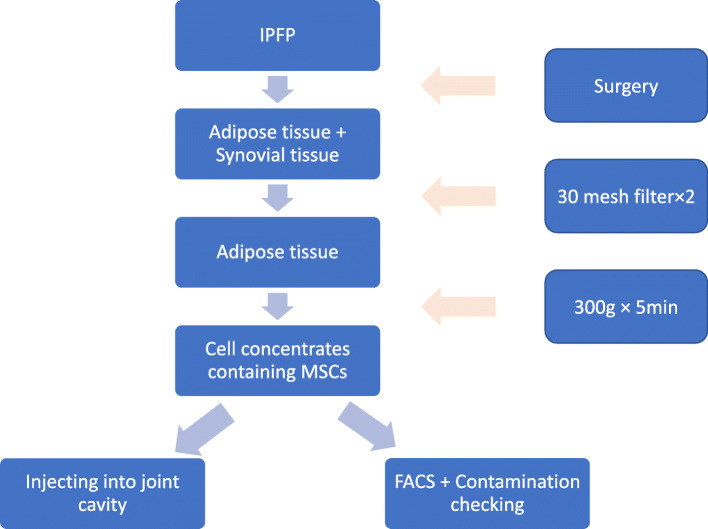
The whole intervention process

All participants in the experiment group received knee arthroscopic therapy by the same surgeon. All the interventions were done in the laminar flow operation room, which was in aseptic condition. After total anesthesia, the surgeon evaluated the medial, lateral, and patellofemoral joint compartments. Then, the surgeon performed one or more of the following treatments: debridement; excision of degenerative tears of the menisci, fragments of articular cartilage, chondral flaps, or osteophytes that prevented full extension. Neither abrasion nor microfracture of cartilage lesion was performed. During the knee arthroscopic therapy, partial IPFP tissue was acquired using a standard motorized shaver system. Briefly, 200 mL of the mixture containing fat, synovial tissue, and 0.9% saline was collected by a sterilized arthroscopic setup of the fat collection system connected with suction apparatus. After being filtered twice using a 30-mesh filter (pore size of 550 μm) to remove the synovial tissue, the mixture was centrifuged at 300 × *g* for 5 min [23]. While the liquid supernatant was discarded, 6 mL of 0.9% saline was used to mix the lipoaspirate to make cell the concentrates. In total, 5 mL of the cell concentrates was injected into the corresponding joint cavity, and another 1 mL was used for identification by flow cytometry [23] and cultured to determine if the cell concentrates were contaminated (Fig. 1).

The cell concentrates were assayed for cell surface protein expression by flow cytometry (FC500, Beckman Coulter, USA). One hundred microliters from 1 mL of freshly isolated cell concentrates was washed twice with phosphate-buffered saline. The antibodies used for the identification of cell concentrates were CD45-FITC, CD44-PE-Cy, CD90-PE, and PE-CD105 (BD Pharmingen, USA). As a negative control, a cell suspension without antibodies was employed following the same procedure. Cell concentrates were incubated with antibodies for 20 min at 4 °C and then resuspended in fluorescence-activated cell-sorting media and analyzed immediately.

#### Control group

All participants in the control group received regular knee arthroscopic therapy after anesthesia. During the arthroscopic therapy, same amount of the IPFP tissue was removed under arthroscope; then, the joint cavity was treated with routine arthroscopic therapy same as the experiment group. After the arthroscopic therapy, 5 mL of 0.9% saline was injected into the treated knee cavity.

#### Concomitant care and interventions

Patients in both groups were restricted from taking corticosteroids and nonsteroidal anti-inflammatory drugs 1 week before surgery. After the surgery, no corticosteroid was injected into the knee articular cavity. During the rehabilitation period, walking and mild activities were not restricted. Subsequently, the gradual resumption of normal sports or recreational activities was allowed. No analgesics or anti-inflammatory drugs were allowed after the treatment. All post-operational treatment and rehabilitation processes were the same in both groups.

### Assessment

All patients were evaluated by an investigator blinded to the group allocation. The Western Ontario and McMaster Universities Osteoarthritis Index (WOMAC) of patients were assessed preoperatively and at 6 weeks, 12 weeks, 6 months, and 12 months after the intervention to evaluate the functional recovery of the knee joint. The secondary objective of the study was to compare the efficacy of the two methods for pain relief using the visual analog scale (VAS) in both static and motion states at pre-operation and 6 weeks, 12 weeks, 6 months, and 12 months after the intervention. The safety of the two methods was assessed by routine blood analysis, hepatorenal function, and ESR and CRP levels at each follow-up.

Knee magnetic resonance imaging (MRI) was conducted to assess the cartilage regeneration at pre-operation and 12 months after the intervention using Magnetic Resonance Observation of Cartilage Repair Tissue (MOCART). All these evaluations were performed by two well-experienced orthopedists; once disagreements existed, a third one would join in.

### Side effects

Adverse reactions were defined as any unexpected events that occurred when the trial was noted and recorded. Serious adverse events (SAEs) were defined as events that were life-threatening or resulted in death, hospitalization, or significant disability.

### Statistical analysis

All statistical analyses were performed with the SPSS software, version 19.0 (IBM, Armonk, NY). All data were expressed as means and standard deviations. Because the primary outcome was the difference in WOMAC score between baseline and 12 months, sample size was set based on the results of our pilot study and previous study [22] (α risk 0.05, power 0.8, 10% losses to follow-up, changes in WOMAC score 15, and SD 8). The required number of patients should be at least 23. To improve the reliability of our study, we increased the sample size to 30 patients per group.The Student *t* tests were used to analyze statistical differences between preoperative and follow-up values of WOMAC scores, VAS scores, and MOCART scores. For all the tests, significance was defined as *P* < 0.05.

## Results

**Fig. 2:**
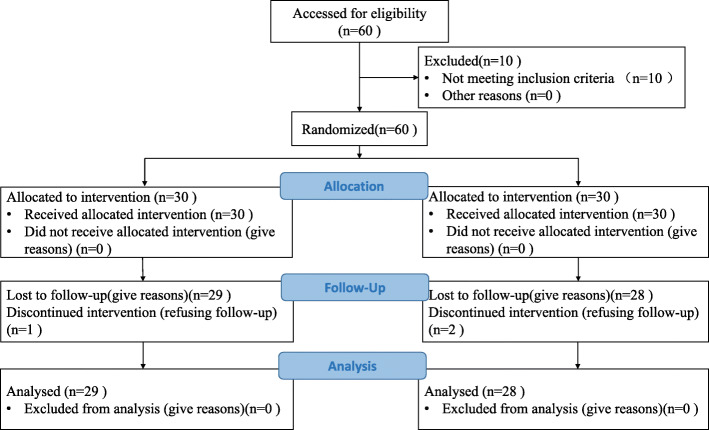
The CONSORT flowchart diagram

**Fig. 3:**
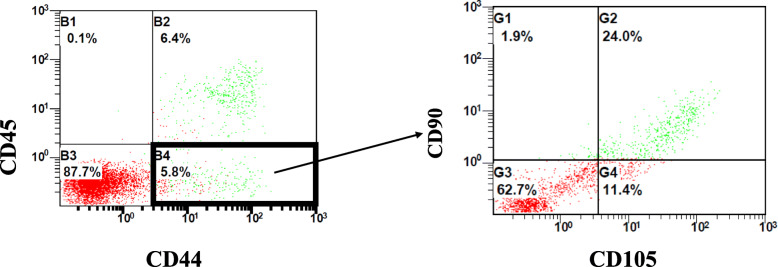
Representative results from the fluorescence-activated cell sorting analysis of freshly isolated cell concentrates for (**a**) synovial markers (CD45-CD44+) and (**b**) mesenchymal markers (CD90 + CD105+). The numbers represent the percentage of gated cells over live cells as the parental population

A total of 30 cases were enrolled in each group. Twenty-nine cases in the experiment group and 28 cases in the control group were followed up (for patients data, see Table 1 and Fig. 2). According to the results of flow cytometry, 1.19 ± 1.17 × 10^8^ cells/ml of cell concentrates in average were harvested from each patient, while 3.91 ± 2.96 × 10^6^ cells/ml of cell concentrates in average were proved to be stromal cells (CD45^-^CD44^+^CD90^+^CD105^+^), with a percentage of 4.38 ± 2.29% in average (Fig. 3, Tables 2 and 3)

**Table 1:** Baseline characteristics of the patients

Characteristic	Experiment	Control	*P* values
Cases (complete F-U)	30 (29)	30 (28)	
Age	52.27 ± 1.17	51.77 ± 7.55	> 0.05
Gender (M/F)	5, 25	8, 22	> 0.05
BMI (Kg/m^2^)	24.19 ± 2.54	23.61 ± 3.04	> 0.05
Kellgren-Lawrence grade			
0	5	4	> 0.05
I	5	7	> 0.05
II	17	16	> 0.05
III	3	3	> 0.05
Type of disease			
Simple knee cartilage lesion	7	6	> 0.05
Meniscus injury	21	23	> 0.05
Ligamentous injury	2	1	> 0.05

Data are presented as mean ± SD

**Table 2:** Arthroscopic harvest of cell concentrates from infrapatellar fat pad

	Total cells ( × 10^8^)	Stem cells ( × 10^6^)	Percentage (%)
Cell concentrates	1.19 ± 1.17	3.91 ± 2.96	4.38 ± 2.29

Data are presented as mean ± SD

**Table 3:** Surface marker of cell concentrates by flow cytometric analysis

Markers	Percentage (%)
CD45^-^	12.50 ± 4.83
CD44^+^	6.70 ± 5.72
CD90^+^	6.80 ± 3.89
CD105^+^	11.02 ± 4.71
CD45^-^CD44^+^CD90^+^CD105^+^	4.38 ± 2.29

Data are presented as mean ± SD of the percentage of cells staining with anti-human mouse IgG antibodies

**Fig. 4:**
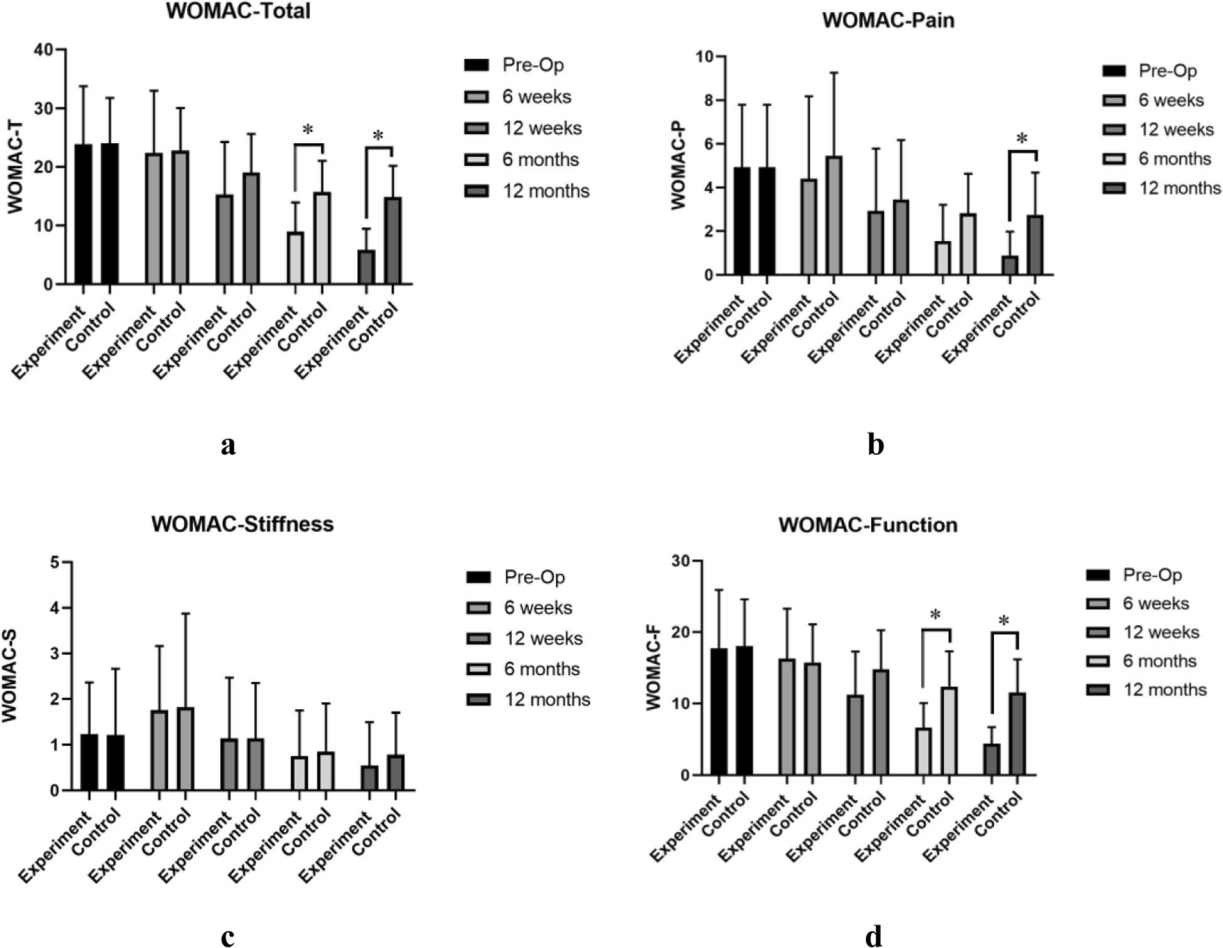
WOMAC scores were evaluated pre-operation, 6 weeks, 12 weeks, 24 weeks, and 48 weeks after intervention. **a** WOMAC total scores. **b** WOMAC pain scores. **c** WOMAC stiffness scores. **d** WOMAC function scores. Experiment group = cell concentrates injection + knee arthroscopy; control group = knee arthroscopy (*p < 0.05)

**Fig. 5:**
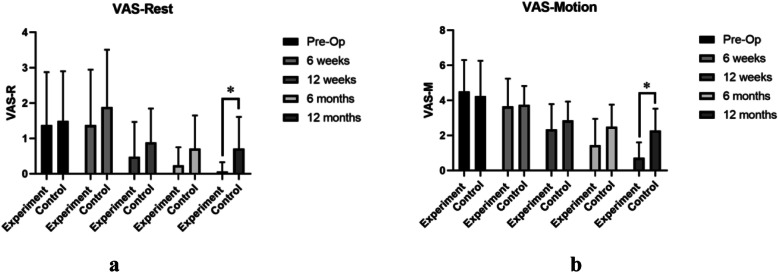
VAS scores were evaluated pre-operation, 6 weeks, 12 weeks, 24 weeks, and 48 weeks after intervention. **a** VAS rest scores. **b** VAS motion scores. Experiment group = cell concentrates injection + knee arthroscopy; control group = knee arthroscopy (*p < 0.05)

**Fig. 6:**
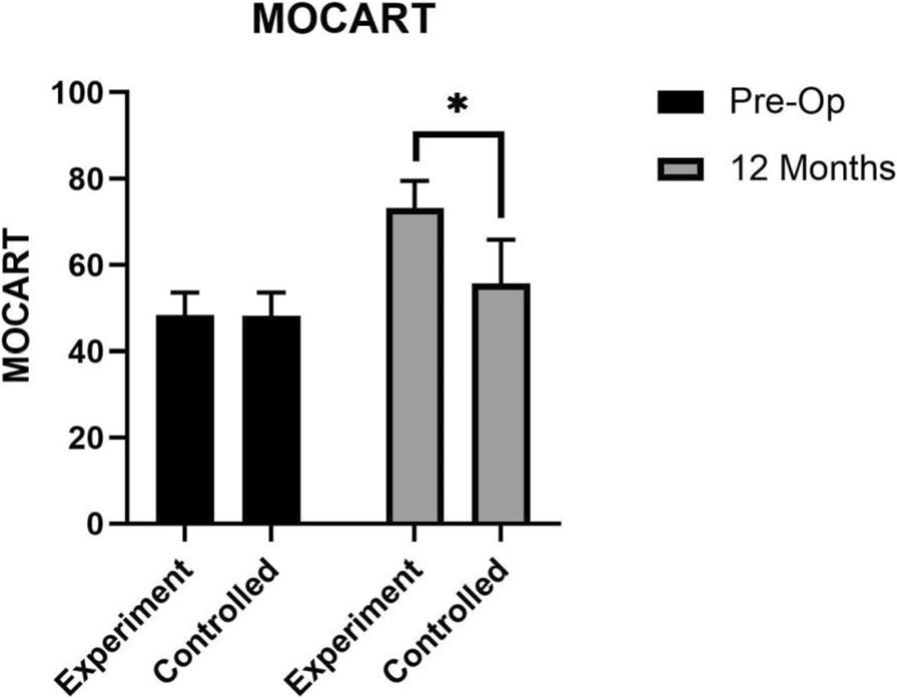
MOCART scores were evaluated pre-operation and 48 weeks after intervention. Experiment group = cell concentrates injection + knee arthroscopy; control group = knee arthroscopy (*p < 0.05)

**Fig. 7:**
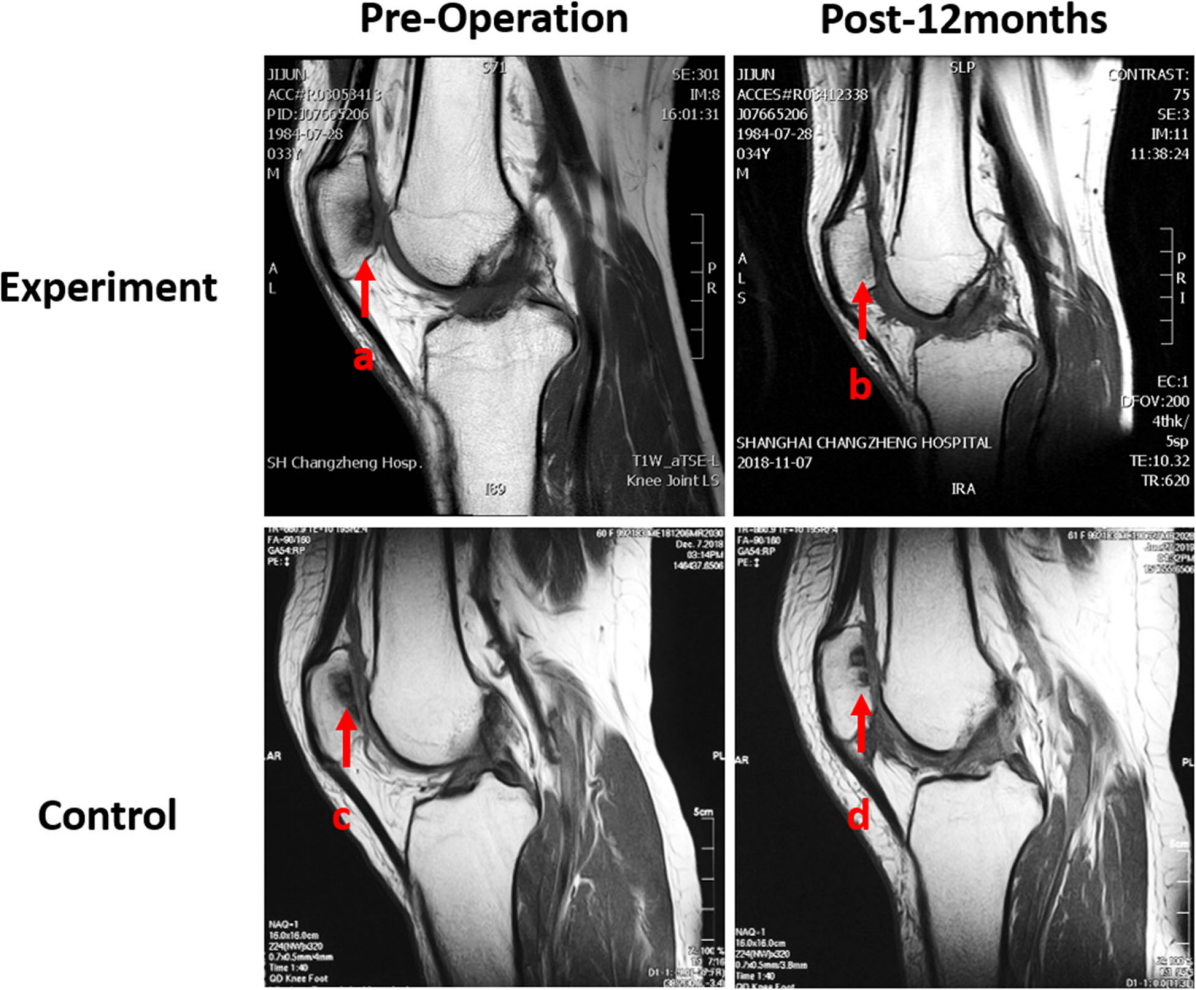
Results of MRI performed preoperatively (**a** and **c**) and 12 months postoperatively (**b** and **d**) on two patients. The experiment group (**a** and **b**) was a 30-year-old male with arthritis in patellofemoral joint. T1-weighted fast spin-echo sagittal view 12 months after surgery showed obvious cartilage reparation with the MOCART score of 80. The control group (**c** and **d**) was a 38-year-old female with approximately same arthritis in patellofemoral joint. T1-weighted fast spin-echo sagittal view 12 months after surgery showed less cartilage reparation with the MOCART score of 55

No significant difference of the average age of patients (52.27 years), VAS, and WOMAC scores were found between experiment group and control group before surgery (*P* > 0.05). WOMAC scores decreased substantially over the follow-up period (Fig. 4). The mean WOMAC total and WOMAC function scores of the experiment group were significantly lower than those of the control group 6 months and 12 months after surgery (*P* < 0.05) (Fig. 4a and d). The mean scores of WOMAC pain of the experiment group were only found significantly lower than those of the control group 12 months after surgery (*P* < 0.05) (Fig. 4b). As for the scores of stiffness, no significant difference was found between the two groups (*P* > 0.05) (Fig. 4c). In the aspect of VAS rest and VAS motion scores, significant decrease in the experiment group was found 12 months after surgery (*P* < 0.05) compared with the control group (Fig. 5). And MOCART scores of the experiment group were also found significantly higher than those of the control group 12 months after surgery (*P* < 0.05) (Figs. 6 and 7). During the follow-up period, no case of articular infection or damage to the hepatorenal function was found (Table 4).

**Table 4:** Outcome measures at pre-operation, 6 weeks, 12 weeks, 6 months, and 12 months

Parameter		Experiment group	Control group	*P* values
WOMAC pain	Pre-op	4.83 ± 2.94	4.96 ± 3.16	> 0.05
	6 weeks	4.57 ± 3.80	5.6 ± 4.24	> 0.05
	12 weeks	3.10 ± 2.95	3.60 ± 3.12	> 0.05
	6 months	1.77 ± 2.06	2.83 ± 2.09	> 0.05
	12 months	1.00 ± 1.26	2.73 ± 2.23	< 0.05
WOMAC stiffness	Pre-op	1.07 ± 1.11	1.33 ± 1.49	> 0.05
	6 weeks	1.80 ± 1.40	1.9 ± 2.02	> 0.05
	12 weeks	1.17 ± 1.32	1.2 ± 1.19	> 0.05
	6 months	0.87 ± 1.14	0.93 ± 1.05	> 0.05
	12 months	0.63 ± 1.10	0.86 ± 0.94	> 0.05
WOMAC function	Pre-op	13.30 ± 9.26	13.73 ± 11.97	> 0.05
	6 weeks	15.93 ± 13.81	14.83 ± 11.00	> 0.05
	12 weeks	11.60 ± 12.11	12.7 ± 10.19	> 0.05
	6 months	6.70 ± 6.96	10.27 ± 8.53	< 0.05
	12 months	4.30 ± 4.04	10.13 ± 8.82	< 0.05
WOMAC total	Pre-op	19.20 ± 11.90	19.90 ± 14.19	> 0.05
	6 weeks	22.30 ± 17.67	22.23 ± 13.14	> 0.05
	12 weeks	15.87 ± 15.13	17.43 ± 12.05	> 0.05
	6 months	9.33 ± 9.44	14.03 ± 9.58	< 0.05
	12 months	5.93 ± 5.84	13.73 ± 9.85	< 0.05
VAS rest	Pre-op	1.40 ± 1.48	1.50 ± 1.41	> 0.05
	6 weeks	1.47 ± 1.61	1.93 ± 1.57	> 0.05
	12 weeks	0.53 ± 1.01	0.97 ± 1.00	> 0.05
	6 months	0.30 ± 0.60	0.77 ± 0.94	> 0.05
	12 months	0.10 ± 0.30	0.73 ± 0.90	< 0.05
VAS motion	Pre-op	4.50 ± 1.76	4.43 ± 2.13	> 0.05
	6 weeks	3.70 ± 1.58	3.8 ± 1.06	> 0.05
	12 weeks	2.60 ± 1.99	2.83 ± 1.05	> 0.05
	6 months	1.67 ± 1.90	2.53 ± 1.25	> 0.05
	12 months	0.77 ± 0.90	2.3 ± 1.21	< 0.05
MOCART	Pre-op	48.33 ± 5.22	48.17 ± 5.68	> 0.05
	12 months	73.45 ± 6.28	52.68 ± 7.26	< 0.05

Data are presented as mean ± SD

## Discussion

In this study, the short-term results are encouraging and demonstrate that knee arthroscopy with IPFP cell concentrates containing MSCs reduce pain and improve function in patients with knee cartilage lesions, especially at 6 and 12 months after surgery. In addition, no significant difference was found in the WOMAC and VAS scores in the early post-surgery stages between the two groups. This may be because the main effect of the MSCs in cell concentrates works to repair the lesion cartilage rather than reducing inflammation in the early postoperative stage [12, 24]. With respect to safety, the therapy did not increase the probability of postoperative infection and had no obvious influence on the hepatic and renal function of patients.

Cartilage lesions have very limited intrinsic healing capacity. Surrounded by fewer vessels, nerves, and lymphoid tissue, cartilage with large lesions undergo repair only with the production of fibrous tissue or fibrocartilage [3]. Therefore, degeneration is likely to occur and can progress to osteoarthritic changes in many cases [25].

MSCs, such as bone marrow mesenchymal stromal cells (BMSCs), synovial-derived MSCs (SDMSCs), and adipose-derived MSCs (ADMSCs), can be extracted from different kinds of tissue, whereas only MSCs derived from bone marrow and adipose can be acquired in large amounts for clinical use [14–16, 26–29]. Compared with BMSCs, ADMSCs have a more active proliferative capacity, and their acquisition causes less damage to the donor zone [24, 30–32]. Meanwhile, studies have also shown that MSCs from different kinds of tissues have significantly different proliferation and differentiation abilities. According to Zhang et al. [33], compared with MSCs derived from the synovium lining of the joint capsule and the synovium surrounding the cranial cruciate ligament, MSCs from the infrapatellar adipose tissue show a higher ability to proliferate and differentiate.

Recent studies suggest that the use of MSCs for treating knee cartilage lesions is a safe way to alleviate pain and improve knee function [34–36]. Toghraie et al. [37] used scaffold-free MSCs obtained from IPFPs in an experimental animal model of osteoarthritis by direct intra-articular injection. Rabbits receiving MSCs showed a lower degree of cartilage degeneration, osteophyte formation, and subchondral sclerosis than control group 20 weeks after surgery. According to a study by Liu et al. [38], it is possible to generate robust, flexible, cartilage-like grafts of scale, indicating that tissues engineered using IPFP-MSCs derived from osteoarthritis (OA) patients could potentially be used to resurface large joint areas damaged by trauma or disease. Skalska et al. [39] showed that MSCs derived from IPFP of rheumatoid arthritis (RA) patients have comparable or slightly stronger osteogenic potential than that from osteoarthritis patients. Koh et al. [40, 41] performed several stem cell injections combined with arthroscopic debridement in patients with knee OA. The short-term results demonstrated that IPFP-MSC therapy with intra-articular injections is safe, aids in reducing pain, and improves function in patients with knee OA. Compared to the in vivo studies mentioned above, our study provided a larger sample and a more controlled experimental design. In addition, MOCART scores were added to evaluate the repair of cartilage lesions, and flow cytometry was used to examine the positive rate of MSCs among the cell concentrates derived from IPFP through knee arthroscopy.

In our study, cell concentrates containing MSCs instead of pure stromal cells were injected into the knee cavity. Compared with obtaining pure stromal cells, the procedure to acquire cell concentrates can be simple and quick, thus, decreasing the operating time and reducing the risk of infection associated with injection. On the other hand, since cell concentrates were derived from IPFP abandoned during regular arthroscopy surgery, it is a way to recycle waste reducing the cost of surgery and increasing patients’ compliance.

Instead of being cultured as in other studies, the cell concentrates containing MSCs were injected into the joint cavity directly during arthroscopy surgery. It is widely believed that culture microenvironment can affect the differentiation of MSCs [27, 28, 42–46], and the culturing process can add the risk of infection. According to the studies, it is known that the proliferation and differentiation of MSCs can be influenced by the paracrine effects of the cytokines and growth factors released by the grafted cells, which can trigger host-associated signaling pathways, increase angiogenesis, and decrease apoptosis. López-Ruiz et al. [47] showed that cells exposed to chondrocyte extracts acquire a characteristic morphological and ultrastructural chondrocyte phenotype, which was confirmed by the increased proteoglycan formation on enhanced collagen II immunostaining. Moreover, chondrocyte extracts induced an increase in mRNA expression of chondrogenic genes such as Sox9, L-Sox5, Sox6, and Col2a1.

According to the identification standards proposed by the International Society for Cellular Therapy (ISCT) in 2006, MSCs should meet the following standards: (1) cell adhesion growth in vitro; (2) positive expression of CD44, CD73, CD90, and CD105 (positive detection rate of flow cytometry > 95%) and negative expression of CD11b, CD14, CD19, CD34, CD45, CD79α, and HLA-DR (the positive detection rate of flow cytometry < 2%); and (3) having the capability to differentiate into osteoblasts, adipocytes, and cartilage cells in vitro in specific culture conditions [21].

It is shown that clinical effect of treating knee cartilage lesion with knee IPFP cell concentrates is positive. However, as the number of cells derived from the process is limited, this type of therapy is only suitable for small areas of cartilage lesions. According to a review by Richter et al. [48], small cartilage lesions (area > 2 cm^2^) are best treated by microfracture and cartilage autotransplantation. As for lesion areas between 2 and 4 cm^2^, cartilage autotransplantation and chondrocyte autotransplantation have similar therapeutic effect. For areas beyond 4 cm^2^, chondrocyte autotransplantation is the best choice. Although the clinical effect of treating knee cartilage lesions with knee IPFP cell concentrates is positive, randomized controlled trials with larger sample size and longer follow-up periods are still needed. So far, there are still several technical obstacles to overcome, such as finding a better method to extract, purify, and maintain multipotential differentiation, promote specificity and function, and enhance the survival and cartilage repair ability of IPFP-MSCs.

The present study has some limitations. First, the number of cells injected into each knee was limited and not equal among patients. Second, the patient sample size was small, and the follow-up period was relatively short. In addition, there were no data collected from routine second-look arthroscopic procedure or pathologic examination. In addition, we lost nearly 5% of our patients when these individuals refused to participate in some parts of the follow-up visits.

In the future, tissue-engineering techniques with cell concentrates containing MSCs hold promise for repairing damaged cartilage within joints. At present, although further randomized controlled clinical trials of this treatment, with more patients and longer follow-up periods, are still needed, this study proposes a new option for clinical treatment of knee cartilage lesions.

## Conclusions

The short-term results of our study are encouraging and demonstrate that knee arthroscopy with IPFP cell concentrates containing MSCs is safe, while reducing pain and improving function in patients with knee cartilage lesions especially 6 months after surgery. Thus, the present study offers a new clinical approach and a series of data for further clinical trials.

## Data Availability

The datasets generated and/or analyzed during the current study are not publicly available because it contains patients’ personal information but are available from the corresponding author on reasonable request.

## Abbreviations

MSC: Mesenchymal stromal cells
IPFP: Infrapatellar fat pad
FBG: Fasting blood glucose
WOMAC: Western Ontario and McMaster Universities Osteoarthritis Index
VAS: Visual analog scale
MRI: Magnetic resonance imaging
MOCART: Magnetic Resonance Observation of Cartilage Repair Tissue
SAEs: Serious adverse events
OA: Osteoarthritis
